# Lessons from the Discovery of Mitochondrial Fragmentation (Fission): A Review and Update

**DOI:** 10.3390/cells8020175

**Published:** 2019-02-19

**Authors:** Dmitry B. Zorov, Ivan A. Vorobjev, Vasily A. Popkov, Valentina A. Babenko, Ljubava D. Zorova, Irina B. Pevzner, Denis N. Silachev, Savva D. Zorov, Nadezda V. Andrianova, Egor Y. Plotnikov

**Affiliations:** 1 A.N.Belozersky Institute of Physico-Chemical Biology, Moscow State University, Moscow 119991, Russia; ivorobjev@mail.ru (I.A.V.); popkov.vas@gmail.com (V.A.P.); nucleus-90@yandex.ru (V.A.B.); lju_2003@list.ru (L.D.Z.); irinapevzner@mail.ru (I.B.P.); silachevdn@belozersky.msu.ru (D.N.S.); plotnikov@belozersky.msu.ru (E.Y.P.); 2 V.I. Kulakov National Medical Research Center of Obstetrics, Gynecology and Perinatology, Moscow 117997, Russia; 3 Department of Biology, School of Science and Technology, Nazarbayev University, Astana 010000, Kazakhstan; 4 Faculty of Bioengineering and Bioinformatics, Lomonosov Moscow State University, Moscow 119992, Russia; zorov@inbox.ru (S.D.Z.); andnadya12@yandex.ru (N.V.A.); 5 Institute of Molecular Medicine, Sechenov First Moscow State Medical University, Moscow 119146, Russia

**Keywords:** mitochondria, fission, division, mitophagy, segregation, asymmetry, cytoskeleton, ultrastructure, quality control, dynamics

## Abstract

Thirty-five years ago, we described fragmentation of the mitochondrial population in a living cell into small vesicles (mitochondrial fission). Subsequently, this phenomenon has become an object of general interest due to its involvement in the process of oxidative stress-related cell death and having high relevance to the incidence of a pathological phenotype. Tentatively, the key component of mitochondrial fission process is segregation and further asymmetric separation of a mitochondrial body yielding healthy (normally functioning) and impaired (incapable to function in a normal way) organelles with subsequent decomposition and removal of impaired elements through autophagy (mitophagy). We speculate that mitochondria contain cytoskeletal elements, which maintain the mitochondrial shape, and also are involved in the process of intramitochondrial segregation of waste products. We suggest that perturbation of the mitochondrial fission/fusion machinery and slowdown of the removal process of nonfunctional mitochondrial structures led to the increase of the proportion of impaired mitochondrial elements. When the concentration of malfunctioning mitochondria reaches a certain threshold, this can lead to various pathologies, including aging. Overall, we suggest a process of mitochondrial fission to be an essential component of a complex system controlling a healthy cell phenotype. The role of reactive oxygen species in mitochondrial fission is discussed.

## 1. Introduction

From the time of its discovery, the mitochondrion was recognized as a highly dynamic structure undergoing changes in shape and volume resulting in a mixed population of long and short mitochondrial fragments within a single cell [1]. However, 35 years ago we reported that the entire mitochondrial population in the cell could be converted into small rounded fragments originating from long filamentous, often branched mitochondrial structures [2]. Although we were able to observe the phenomenon of global mitochondrial fragmentation in the cell in response to a large number of drugs having both mitochondrial and non-mitochondrial targets [3,4], at that time it was not possible ascribing this phenomenon to any known physiological situation. Later, mitochondrial dynamics became the subject of a more comprehensive study (reviewed by [5,6]). Nowadays, the global fragmentation of mitochondria (mitochondrial fission), among other functions, is attributed to a cascade of reactions leading to pathological phenotypes and cell death [7,8,9,10], which puts this phenomenon in the focus of a vast number of studies [11,12,13,14,15]. 

Since details of the intricate mechanism of mitochondrial fission have been frequently and comprehensively explored [16,17,18,19], here we will review and update the reader on research of this phenomenon which may help to clarify some hidden elements of its mechanism as well as its role in mitochondrial and cellular physiology. Besides, we will briefly present a general picture of changes associated with mitochondrial fragmentation division/fission/scission.

A master regulator role in mitochondrial fragmentation has been assigned to dynamin-related proteins—Dnm1p in yeast and Drp1 in mammals. Mitochondrial division begins with the recruitment of cytosolic Drp1, which self-assembles into polymers. GTP-dependent oligomerization of Drp1 drives a limited constriction of a specific mitochondrial locus in an energy-dependent way (with GTP as an energy source) forming a belt, compressing the site where mitochondria division will occur [20]. It is important that the constriction occurs at mitochondria–ER contact sites [21] in a Ca^2+^-dependent mode [22]. 

There is data that the initial signal for mitochondria division comes from a site of mtDNA replication located close to the mitochondria–ER contact site [23]. The next step of the mitochondrial division is the recruitment of adapter proteins (MFF, MiD49, and MiD51 [24]) and outer mitochondrial membrane-anchored protein Fis1 [25]. The last stage of the mitochondria division is the recruitment of actin and myosin IIa, which provides the mechanical force to drive further constriction [26,27]. Besides, phospholipids of the outer mitochondrial membrane are involved in the final stage of the fission machinery [28], forming a narrow hauling between two mitochondrial compartments. Further, this bridge is cut either with the assistance of another member of dynamin family, Dnm2 [29] when the mitochondrial radius is below 50 nm, or without the participation of Dnm2 when the mitochondrial radius is up to 250 nm [30]. Thus, the consortium of cytosolic proteins is involved in the process of mitochondrial fragmentation [31].

## 2. Fission as a Possible Means of Segregating and Deleting of Damaged Mitochondrial Compartments

**Figure 1:**
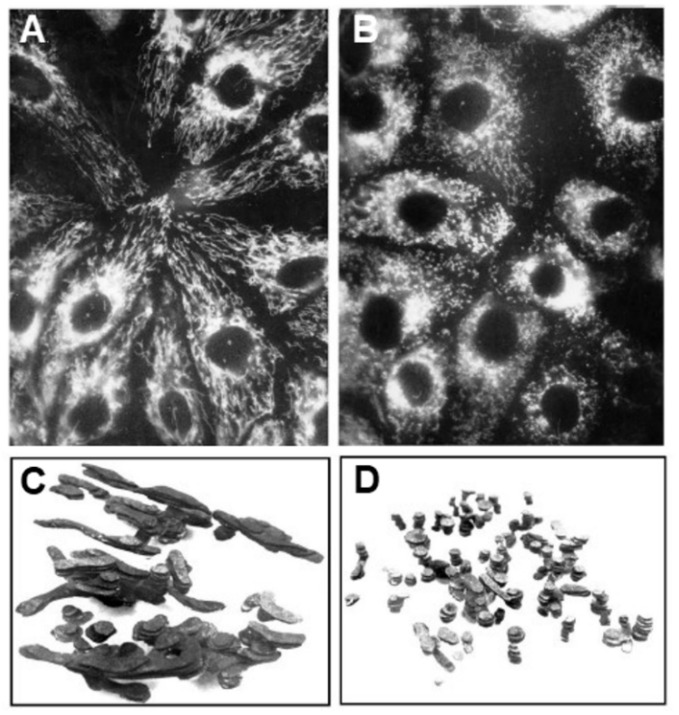
Fragmentation of mitochondrial reticulum in pig embryo kidney epithelial cells. (**А**) fluorescent microscopy of control culture loaded with rhodamine 123 (10 µM), (**B**) the same culture loaded with rhodamine 123 after rotenone (2 µM) treatment for 6 h, and (**C**,**D**) three-dimensional reconstruction made from electron microscopic serial section images for (**A**) and (**B**), respectively. (From [41] with permission).

Diazepam was the first compound identified to induce mitochondrial fission, also inhibiting cells respiration at relatively high concentration [2]. Later, a long list of global fission inducers has been compiled. In this list, the dominant positions were occupied by respiratory inhibitors like rotenone (Figure 1A,B), antimycin A, cyanide, azide, oligomycin, and uncouplers [3,4], all of which diminish the mitochondrial inner membrane potential (Δψ). The electron microscopic study confirmed initial light microscopy observations that this fission was associated with chopping mitochondrial filamentous bodies into separate independent fragments ([2], also see reconstruction in Figure 1C,D). 

**Figure 2:**
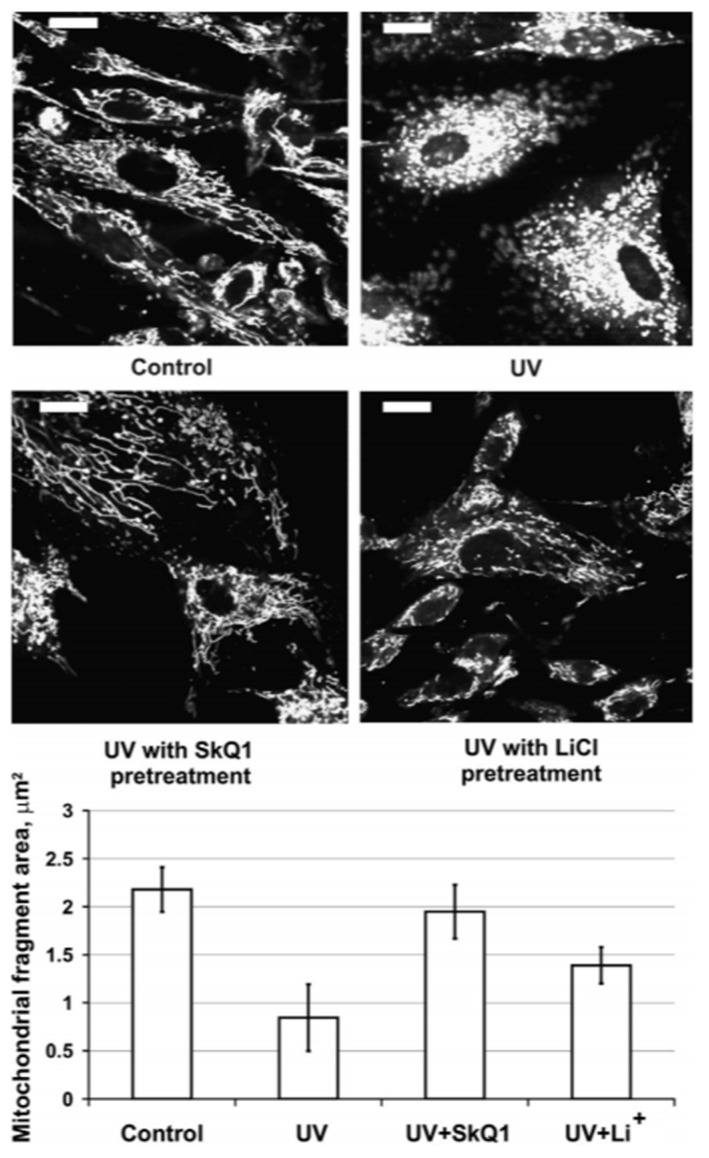
Mitochondrial fragmentation in 60-s UV-exposed fibroblasts (**upper left**, control culture; **upper right**, the same culture after exposure to UV). TMRE (200 nM) staining. The cells pretreated with 120 nM SkQ1 (for 5 days, **bottom left**) are fully protected while with 3 mM LiCl (**bottom right**) partially protected from UV-induced fragmentation. Bar, 20 μm. Diagram illustrates alterations of average mitochondria fragments size (area occupied by a single mitochondrial fragment) under these particular conditions. (From [38] with permission.)

**Figure 3:**
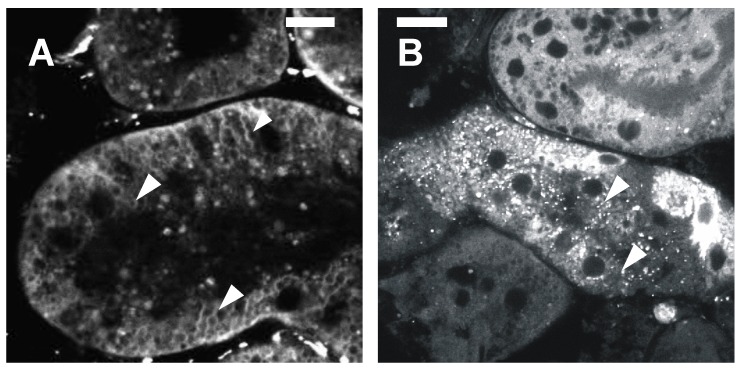
Mitochondria structure revealed by a membrane potential probe (TMRE) in vital rat kidney slices. (**A**) Control kidney slice; (**B**) the slice made from the kidney exposed to ischemia/reperfusion. Bar, 1 µm. (From [39] with permission.)

Mitochondrial fragmentation or, as we later called it, “thread-grain transition” [32], can be chemically-induced in cell culture [2,4,33]. The algorithm for estimating the size distribution of the mitochondrial population within a cell has been suggested [34,35], and we successfully modified and used it for the evaluation of a 3-D steady-state of mitochondrial reticulum [36]. Quantitative evaluation of a level of mitochondrial fragmentation is applicable for widefield fluorescent microscopy of cells attached to a substrate. Figure 2 demonstrates global mitochondrial fragmentation in the culture of the tubular epithelial cells under oxidative stress caused by hypoxia/reoxygenation or UV exposure [37,38] (Figure 2, upper level). These facts became a milestone demonstrating that the phenomenon of a global mitochondrial fragmentation may be observed without chemical treatment. Additional evidence that mitochondrial fission plays a physiological role came from experiments on the intact organ when global mitochondrial fission was observed in cells of a kidney exposed to ischemia/reperfusion [39] (Figure 3 with arrowheads pointing to elongated mitochondria in A and broken mitochondria in B). Thus, the phenomenon of a global mitochondrial fragmentation has been observed both at the cellular and organ levels. 

The core components of mitochondria scission machinery are specific evolutionary conserved proteins, mostly GTPases residing in the outer membrane, intermembrane space, and the inner membrane. They rearrange and remodel these mitochondrial compartments to separate the mitochondrial fragment from the paternal mitochondrial body [13,16,17,18,19,40,41]. There is a consensus that the shift towards either of these two morphological states (thread or grain) can be achieved by specifically blocking one of two processes, resulting in either fission when fusion apparatus is retarded or fusion when fission machinery is blocked. Such changes between different states can be achieved indirectly by modulating the levels of PINK1 and Parkin proteins, involved in mitochondrial quality control mechanisms. Overexpression of Parkin results in a mentioned shift of a balance between fission and fusion and elimination of defected mitochondria, thereby enriching cells for wild type mtDNA and restoring mitochondrial enzymatic activity [42]. Overexpression of PINK1 can rescue mitochondrial morphology and ameliorate ATP levels, cell integrity, and survival of the organism [43]. Ultimately, it suggests that modulation of Parkin and PINK1 expression can ameliorate certain mitochondrial diseases.

To understand the possible intrinsic mechanism of global mitochondrial fission, we must look at the whole design of the mitochondrial architecture in the cell. In a majority of cells, mitochondria form a reticular structure either by organizing a unified tree with continuous matrix [44] (as in fibroblasts or epithelial cells) or with mitochondrial compartments having separate matrices (as in striated muscles, including cardiac myocytes, where mitochondria form a reticulum consisting of head-to-head mitochondria joined by intermitochondrial junctions [45]). The advantage of such unification may be in the ability of such structures to form a networked electric power plant. It is able to conduct the electrical form of transmembrane proton motive force to the regions experiencing an energy deficit either along extended coupling of the inner membranes (as in the case of fibroblasts and epithelial cells) or along many separate mitochondria unified by electrically permeable intermitochondrial junctions (as in the striated muscle) [46,47,48,49]. 

On the other hand, mitochondrial unification is a double-edged sword since possible accumulation of oxidized mitochondrial components (proteins, phospholipids, and DNA) can jeopardize not only the existence of the whole mitochondrial tree, but the cell fate as well, because a single damaged mitochondrion can generate a death signal for the host cell [50]. 

On the other hand, mitochondrial fission may help ensure the survival of mitochondrial DNA by distributing DNA copies over isolated mitochondrial fragments. Specifically, in this context, it looks attractive to assume that the most essential thing for the mitochondria functioning may be the maintenance of the stability of mitochondrial DNA. The latter is hidden behind two mitochondrial membranes with one carrying very high membrane potential (negative inside) prohibiting the passive inward transport of anions including nucleic acids thus making a membrane potential a vital requisite for normal mitochondria and cell functioning [51]. We can see an analogy between the mitochondrial fission/fusion processes and the nucleic acid base excision repair pathway. In the latter, the oxidized base is recognized, excised, and decomposed with further creation of a single-nucleotide repair patch, thus returning to the original intact DNA structure [52]. Similarly, mitochondrial fission may act like an excision step and later stage of fusion may be considered as a return to the original intact organelle, however, accompanied by the prohibition of the participation of the damaged fragment in the organization of the novel mitochondrion. Based on this model, it looks reasonable to suggest that mitochondrial fragments should carry different membrane potential values since one or more fragments have undergone oxidative damage, and this suggestion was confirmed [53], thus showing functional asymmetry of fission events. Moreover, it has been shown that the fusion step which follows after the fission is forbidden for mitochondria carrying low membrane potential [53], although the magnitude of this membrane potential threshold remains unknown. 

**Figure 4:**
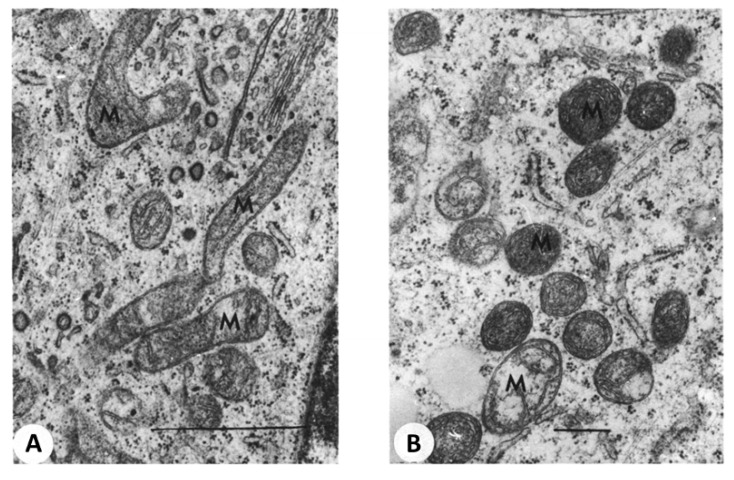
Electron microscopy of ultrathin sections of cultured pig embryo kidney epithelial cells. (**A**) control culture; (**B**) after diazepam treatment (150 µg/mL, 20 h). M-mitochondria. Bar, 1 µm). Note that in exposed cells (**B**), mitochondria with dense matrix coexisted with swollen mitochondria. (From [2] with permission.)

Recently we discussed the issue of biological asymmetry during division including mitochondrial division [54]. Apparently, mitochondrial fragmentation, occurring in response to the oxidative challenge, leads to heterogeneity in the mitochondrial population thus shifting a balance between normal and low-functional mitochondria. Figure 4 demonstrates mostly elongated mitochondrial profiles with uniform ultrastructure in control cell culture (A), and round profiles of mitochondria with two distinct conformations after exposure to a drug (B). 

**Figure 5:**
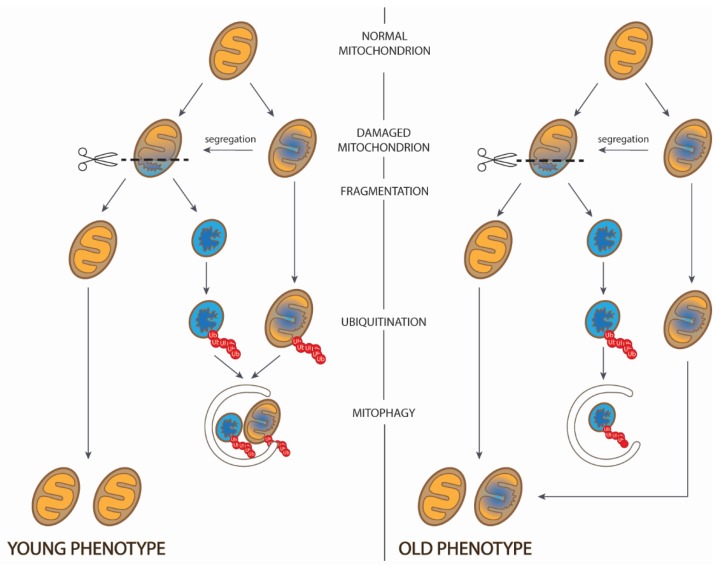
Mitochondrial fragmentation as a determinant of young and old mitochondrial phenotypes. First step represents normal uninjured mitochondrion; in the next step mitochondrion with damaged structures are shown by blue; third step represents segregation of normal and damaged mitochondrial compartments; next step is fragmentation of a mitochondrion, resulting in appearance of normal and abnormal mitochondrial populations (note that partially damaged mitochondrion in the second step can bypass step III and in young phenotype is directed to the lysosome); and then abnormal mitochondrion is ubiquitinated (shown by red chains) and directed to the lysosome. Thus, the difference in young and old cell phenotypes is in abundance of the mitochondrial population with impaired structures in old phenotype (modified from [54]).

The mission of autophagy (mitophagy) is to reverse the balance to a normal one by the disposal of dysfunctional (or low-functional) mitochondria. These two elements (mitochondrial fragmentation and mitophagy) are the essence of the mitochondria quality control machinery which goal is to maintain a healthy (young) mitochondrial phenotype. What is important, after fragmentation mitophagy eliminates mitochondria harboring mtDNA mutations, and the remaining mitochondrial fragments undergo fusion. Thus, fission followed by mitophagy controls the process of reducing heterogeneity of mtDNA and reducing mitochondria heteroplasmy levels, thus pursuing the above-mentioned goal to maintain the native integrity of the mtDNA [55]. The impaired machinery of fission or/and mitophagy leads to the appearance of unhealthy (old) mitochondrial phenotype (Figure 5).

However, the mitochondrial quality control system is not limited to the delicate management of the mitophagy process. In a recent review, mitochondrial quality control mechanisms were deservedly called multi-tiered, operating at the protein, organelle, and cell levels [56]. Indeed, these mechanisms include both the removal of damaged organelles and mitochondrial biogenesis providing a constant flux between degradation and biogenesis. In addition, it includes the homeostatic regulation of mitochondrial turnover on three levels: protein, organelle, and 3-D network level. There is an apparent cross-talk between mitochondrial and other quality control pathways, including mitochondrial unfolded protein response, proteases, ubiquitin-proteasome system and formation of mitochondria-derived vesicles [56].

Autophagy is believed to be a terminal step in the mitochondria life cycle, representing one of the possible mechanisms of mitoptosis, in which the permeability transition may play a primary role [57,58,59,60]. A more exotic means of elimination of mitochondria and their content takes place when they are ejected from the cell into the extracellular medium after fragmentation [61,62,63,64], although the mechanism of such ejection remains unknown. It seems plausible to suggest that fission is necessary for segregation of mitochondria in order to delete an unwanted mitochondrial compartment while the fusion is aimed at organizing healthy mitochondria into a network. Another opinion considers the fusion of mitochondria to serve for selective mixing and unification of mitochondrial compartments, which is essential for the inheritance and maintenance of the mitochondrial genome [65]. Depolarized mitochondria incapable of fusion likely become a target for autophagy [53,65]. The observation that fission inhibition results in the decreased mitochondrial autophagy and accumulation of oxidized mitochondrial products in the cell [53] supports the segregation function of fission. 

**Figure 6:**
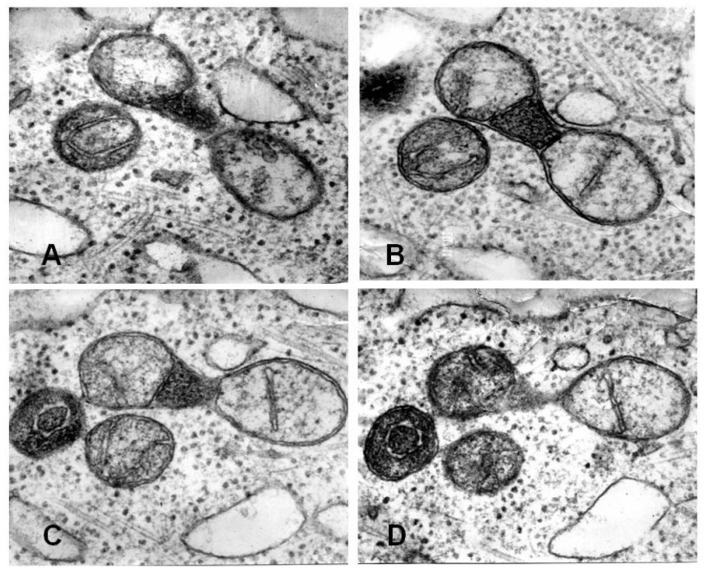
Electron microscopy of four consecutive serial sections (**A**–**D**) over mitochondria of pig embryo kidney epithelial cells exposed to 140 µM diazepam for 16 h. Note the dumbbell-like shape of the central mitochondrion, which contains three separate compartments of different configuration. All three compartments are enclosed within the common outer membrane. Used methods as described in [2].

Detailed analysis of the mitochondrial fission process shows that fragmentation of the inner membrane space precedes the breakage of the outer membrane [13]. This seems reasonable since segregation of the matrix compartment destined for elimination is a prerequisite for its spatial and functional separation from the remaining (undamaged) matrix part. It can prevent the leakage of mitochondria damaged components into the cytosol and will preserve the sealing properties of the inner membrane. One can expect that the situation where different mitoplasts share one common outer membrane must not be unusual. Figure 6 demonstrates that this is exactly the case when fission of mitochondria is launched. In this figure, within a shared outer membrane, there are three mitoplasts carrying different conformations, i.e., having different energization.

## 3. Are Reactive Oxygen Species (ROS) Involved in Mitochondrial Fragmentation?

**Figure 7:**
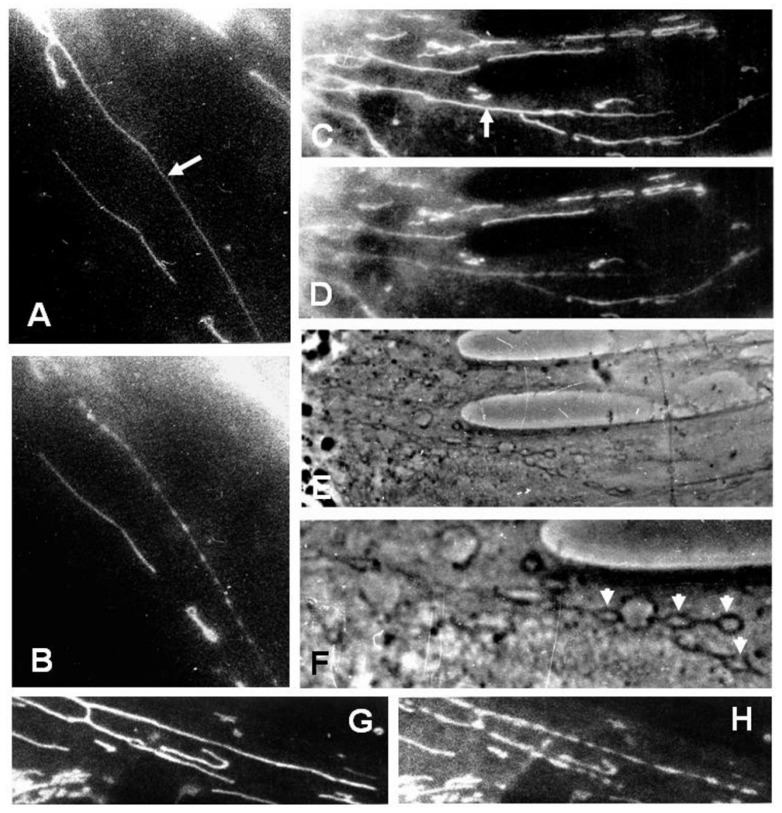
Photodynamically-induced mitochondrial fragmentation in human skin fibroblasts loaded with rhodamine 123. (**A**–**D**) mitochondrial fragmentation caused by a focused laser beam (the locus of irradiation is indicated with an arrow; Ex = 543 nm, 0.1 W, exposure time 1/30 s). (**A**,**C**) Before and (**B**,**D**) 30 s after the irradiation. (**E**,**F**) Phase contrast picture of the cell region in (**C**) and (**D**) under low and high magnification; arrowheads point to the local expansions of mitochondrial filaments resulting in the formation of a “reading glass” mitochondrial shape. Note that mitochondrial dye in (**D**) has been significantly depleted over the entire visually fragmented filament. (**G**,**H**) Mitochondrial shape changes after continuous illumination of the cell with blue light (1 min under the Zeiss microscope); before (**G**) and 1 min after the illumination (**H**). Used methods as described in [2].

Earlier, we postulated that ROS could be involved in the mitochondrial fission [32]. Later, we found that mitochondria-targeted antioxidants prevent mitochondrial fragmentation caused by the oxidative stress [38] and this, as well as some other indirect data, demonstrates the critical role of ROS in mitochondrial scission. It has been also shown that direct exposure of cells to hydrogen peroxide causes either transient mitochondrial fragmentation when the oxidative challenge was transient, or significant changes in mitochondrial morphology and content when the oxidative stress was persistent [66]. In two different sets of experiments, we observed the process of mitochondrial fragmentation induced by photodynamic processes. Due to the existence of the mitochondrial and cell membrane potential, certain cationic fluorescent dyes are accumulated in the mitochondrial matrix to a very high concentration, exceeding that in the cytosol by more than three orders of magnitude. After excitation of these dyes with light, ROS are generated inside mitochondria and can cause severe damage (such as the induction of the permeability transition [57,58]) or moderate damage (as at frequently reversible mitochondrial uncoupling [46,47]). In rhodamine-123 stained cells, we observed fragmentation of the mitochondrial filament occurring within a few seconds after either exposure to a short pulse (1/30 s) of green laser or constant illumination with the violet-blue light of the fluorescent microscope (400–475 nM). Both types of irradiation generate ROS (Figure 7A,B; the arrow points to a targeted mitochondrion). Also, at the high magnification of the light microscope, we were able to detect an intermediate step of filamentous mitochondria fragmentation in which local mitochondrial expansions are interconnected to form a “reading glass/dumbbell-like” pattern of mitochondria which further evolves into a set of small, barely-recognizable mitochondrial fragments. Figure 7 shows mitochondria in the fibroblast (C) later exposed to a local laser excitation light causing mitochondrial fragmentation (D–F).

It is obvious that multicomponent mitochondrial fission machinery is designed in a way to provide multiple controlling elements in order to maximally prevent spontaneous mitochondrial fission ultimately ending in unwanted cell death. Essential time is needed for the recruitment of entire proteinaceous machinery to complete a multistep fission process (starting from intermitochondrial segregation following by formation of the septum (as in Figure 6) and further separation of mitochondrial fragments into those sentenced to either death or survival. However, the entire mitochondrial fission can take seconds when high levels of ROS become a challenge, apparently accelerating or bypassing partial reactions of the process. Indeed, the example given in Figure 7 takes these seconds to accomplish mitochondrial fragmentation caused by the photodynamic process. Earlier, it has been demonstrated that oxidative stress causes activation of PKCδ leading to Drp1 phosphorylation and translocation of the Drp1/PKCδ complex to the outer mitochondrial membrane, where Drp1 binds to Fis1 [67]. This is nothing more than an indication that these mechanisms of mitochondrial fragmentation may be either different [68] or mitochondrial division and fragmentation/fission are not identical processes.

## 4. The Assumption of the Existence of an Intramitochondrial Skeleton

**Figure 8:**
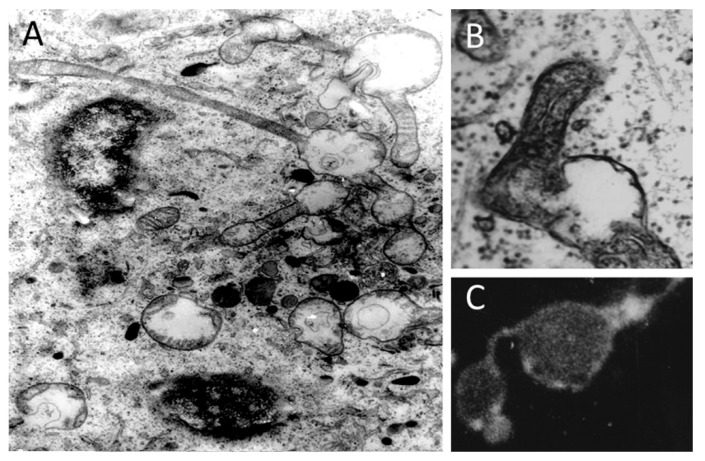
Local mitochondrial swellings *in situ*. (**A**) Electron microscopic picture of a cell from a kangaroo rat kidney epithelium exposed to high hydrostatic pressure (100 mPa). Note multiple local mitochondrial expansions (local mitochondrial swellings) over a single mitochondrial filament with continuous matrix; electron microscopic and fluorescent microscopic evidence (**B**,**C**), respectively of a local swelling of mitochondrial filaments in pig embryo kidney epithelial cells after exposure to diazepam. Rhodamine 123 staining in C; note that mitochondrial stain is localized in spots adjacent to the membrane. Used methods as described in [54].

As we indicated, the mitochondrial membrane potential should be uniform over one continuous inner membrane [46,47]. Theoretically, when there is a change in mitochondrial energization, the configuration of the whole mitochondrial compartment must change, and there is electron microscopic evidence to support this (Figure 2). On the other hand, although light microscopy does not permit us to make a conclusion, one intermediate step in mitochondrial fragmentation may involve local mitochondrial swelling with further scission of mitochondria into fragments (Figure 7). By electron microscopic analysis, we were able to detect the dumbbell-like mitochondria captured in an early stage of mitochondrial division. The observation of dumbbell-like mitochondria seems to conflict with the general opinion that the mitochondrion behaves like a real osmometer since within a separate insulated compartment the osmotic force created by its content must be single-valued. If this were true, then we would not be able to detect local changes of the configuration within a single mitochondrion and local swellings should not occur. However, Figure 8 demonstrates that filamentous mitochondria may undergo local expansions along their length. This result can be explained only if one assumes the existence of some elements (probably of cytoskeletal origin) which locally constrain the expansion of mitochondria. Uneven changes of mitochondrial ultrastructure were observed only in a living cell in situ; such changes were never observed in vitro in isolated mitochondria, which do behave as real osmometers and swell or shrink equally along their volume. 

Perhaps the first report, albeit not fully confirmed by subsequent evidence, on mitochondrial volume changes caused by non-osmotic forces with the assumption of the presence of contractile proteins in mitochondria was a short communication published by Ohnishi and Ohnishi in 1962 [69]. The concept of the existence of contractile proteins in the mitochondria was also supported by one of the most known biochemists, Albert Lehninger [70]. In addition, a few years later it was emphasized that “an explanation of changes in mitochondrial volume exclusively in terms of osmotic phenomena is far from adequate” [71]. Such inconsistency between ultrastructural changes and functionality of mitochondria has been later forgotten in parallel with a loss of excellent old school of electron microscopists, which in combination with superior bioenergetic school, used to give information on the relationship of mitochondrial structure and functions. However, we must admit that there was growing evidence on the interaction of mitochondria with other cellular components including cellular cytoskeleton. In 1978, the association of mitochondria with microtubules was reported [72] with subsequent confirmation of the role of tubulin in the regulation of mitochondrial activity [73,74,75]. With the discovery of rhodamine 123 allowing visualization of fluorescing mitochondria in a cell [76], it became possible to observe the intracellular distribution of these organelles relatively to cytoskeletal elements obviously pointing to their interaction. Mitochondria were found to tightly associate with intermediate filaments [77], which can be distinguished biochemically and immunologically and we can classify several types of intermediate filaments known that they interact directly or indirectly with mitochondria (reviewed in [78]). These include vimentin, keratin and desmin filaments, neurofilaments, and glial acidic protein filaments. It is important that intermediate filaments are highly susceptible to proteolysis, especially the Ca^2+^-dependent one. This proteolytic mechanism is thought to have a regulatory role in changes of shape and locomotion thus making intermediate filaments integrators of cellular (and mitochondrial) space [79]. Apparently, regulation of the mitochondrial shape [80] providing a balance between fragmentation and fusion, ultimately modulates the energy balance of a cell [81]. Besides (or due to) that cytoskeletal elements act as organizers of the cellular space, they perform a large number of specific functions participating in cell growth and death, differentiation, signal transduction, and motility [78]. For instance, vimentin filaments were found to regulate mitochondrial membrane potential [82] and to exert a strong influence on the mechanisms regulating mtDNA duplication and afford tolerance to oxidative stress [83]. Similar effects of desmin on the respiratory capacity of mitochondria, metabolic channeling, compartmentation, and energy transfer networks were reported [84,85,86].

In all aforementioned schemes, intermediate filaments and microtubules have been designated as extramitochondrial structures. It is clear that membrane cellular elements (e.g., endo-/sarcoplasmic reticulum) and cytoskeleton tightly interacting with mitochondria in the cell can restrain changes in mitochondrial shape and somehow maintain the stability of mitochondrial volume. However, local mitochondrial swelling in situ which we observe as partial enlightenment of the mitochondrial matrix (an example is given in Figure 8B) cannot be fully explained by factors external to mitochondria such as ER [87]. The reasonable explanation of this is an assumption of the presence of intramitochondrial 3-D frame, which may locally respond to the structural and functional challenges.

The idea of an intramitochondrial skeleton serving as a solid organelle framework or armature has been already discussed in the 1980s [88] after detection of intramitochondrial filaments [89] and intracristae helical structures [90]. This idea is still under debate. Another, although indirect evidence of structural organization of mitochondria is the result of the analysis of mitochondrial deformation demonstrating significant structural anisotropy between the orthogonal short axes (i.e., width and depth) [91]. Admitting the scarcity of available information, we would like to notice that although the presence of actin in the nucleus was first suggested almost half a century ago [92], after a long period of skepticism, this has only recently been demonstrated directly (reviewed in [93]). A similar fate can befall the intramitochondrial “mitoskeleton” since recent findings support the existence of intramitochondrial cytoskeleton at least in the form of γ-tubulin [93]. Immunoelectron microscopy confirmed that γ-tubulin is a mitochondrial protein, which, when diminished, causes cytochrome C release from mitochondria. In spite of the great similarity of the mitochondrial proteome to that of some bacteria, most mitochondria lack FtsZ playing a role of a major cytoskeletal element in bacterial division [94], and possibly γ-tubulin plays a similar role in mitochondrial division [93]. It seems very attractive to speculate that this protein, and/or some other cytoskeletal elements are involved in the intramitochondrial segregation of waste products eventually leading to asymmetric mitochondrial division.

## 5. Conclusions

Mitochondrial shape is the result of the balance of two opposing processes: fragmentation (fission) and assembly (fusion). We speculate that mitochondrial fission is a form of intramitochondrial compartmentalization which allows segregation of mitochondrial components (proteins, lipids, and DNA) destined to be mitochondrial waste ready for decomposition with a principal role of the cytoskeleton in this “clean-up” process. The well-organized decomposition and removal of unwanted/damaged mitochondrial components is a part of the normal turnover, which includes a poorly understood process of mitochondrial mixing and segregation. Additional studies are still needed to prove the universality of mechanisms of mitochondrial fission/scission (fragmentation). Technically, it is not so easy to prove the fragmentation in striated muscle, where the mitochondrial reticulum is formed by small mitochondrial bodies joined by intermitochondrial junctions [45] forming an electrically coupled unit [46]. For irrefutable proof, 3-D reconstruction of mitochondrial content of the striated muscle cell has to be done at high resolution. Currently, researchers limit themselves to the description of the increased level of fission proteins or reduced level of fusion proteins [95]. Others use electron microscopic images of single ultrathin sections and describe changes in the size and number of mitochondria there [96]. This is not enough since morphological changes of mitochondria not necessarily match the changes in the levels of proteins involved in mitochondrial dynamics [97]. However, a limited number of studies such as those had used models of heart failure, chronic contractile activity, or heat exposure provided some evidence of the increased level of mitochondria fragmentation concordant with changes of fission/fusion proteins [97,98,99]. The same problem exists for mitochondria in the midpiece of the spermatozoon, which are also organized into an electrical circuit [100], possibly by means of intermitochondrial cement [101]. One can speculate that in these sophisticated systems, the elimination of unwanted (damaged) mitochondria may be preceded by the electrical decoupling of a particular mitochondrion. Nevertheless, in these and other cell types, including cardiac myocytes, our knowledge of the mitochondrion as a structurally autonomous entity is incomplete, albeit the progress is impressive [102,103].
